# Differentiation between intestinal Behçet’s disease and Crohn’s disease based on endoscopy

**DOI:** 10.3906/sag-1807-67

**Published:** 2019-02-11

**Authors:** Jing-Fen YE, Jian-Long GUAN

**Affiliations:** 1 Department of Immunology and Rheumatology, Huadong Hospital Affiliated to Fudan University, Shanghai P.R. China

**Keywords:** Intestinal Behçet’s disease, Crohn’s disease, endoscopy, differential diagnosis

## Abstract

**Background/aim:**

Differentiating intestinal Behçet’s disease (BD) from Crohn’s disease (CD) is highly challenging, as they often mimic each other in terms of clinical manifestations. Endoscopy is an important modality for distinguishing bowel lesions. The study was designed to identify clinical manifestations that are easily confused and to evaluate the efficacy of endoscopy for distinguishing intestinal BD from CD by several overlapping signs.

**Materials and methods:**

The data from 111 patients with intestinal BD and 81 patients with CD were retrospectively analyzed. Logistic regression was applied to establish a prediction model based on endoscopic findings for the differential diagnosis. The diagnostic efficacy of endoscopy was verified using the area under the receiver operating characteristic (ROC) curve.

**Results:**

Among intestinal BD patients mucocutaneous lesions were the leading clinical manifestations. Gastrointestinal symptoms were common in CD but were rare in intestinal BD (P < 0.001). CD patients with moderate-to-severe activity were more common than intestinal BD patients presenting with equivalent activity (P < 0.05). Independent factors that distinguished intestinal BD from CD were solitary ulcer in the ileocecal area (P < 0.001), perianal abscess (P = 0.049), single segment (P < 0.001), round intestinal ulcer (P = 0.013), intestinal obstruction (P = 0.035), and fistula (P < 0.001). The scores ranged from –2 to 3. The area under the ROC curve was 0.874 (95% CI: 0.823–0.926) (P < 0.001). With a score of 1.5 as the diagnostic cutoff value, the sensitivity and specificity were 76.3% and 80.6%, respectively.

**Conclusion:**

Mucosal injuries were rarer in patients with intestinal BD than in those with CD. The differentiation model combining several endoscopy features appeared to be reliable for distinguishing between intestinal BD and CD.

## 1. Introduction

Behçet’s disease (BD) is a systemic vasculitis that causes inflammation of all sizes of vessels, with involvement of several organs. Its typical manifestations include recurrent oral ulcers combined with genital ulcers and skin lesions, and it frequently involves various other organs, including the eyes, central nervous system, and gastrointestinal tract (1). Intestinal BD is diagnosed when there are documented ulcerative lesions in the terminal ileum or alimentary tract and clinical manifestations that meet the diagnostic criteria for BD (2). The incidence of intestinal BD shows a wide variation across geographies, with low relative frequency in Turkey (1% of BD patients), moderate frequency in the China (17% of BD patients), and high frequency in Japan (50% of BD patients) (1). The real frequency of intestinal BD might be higher due to the absence of gastrointestinal manifestations in considerable numbers of patients (3). Crohn’s disease (CD) is a chronic relapsing, transmural inflammatory disorder that most commonly affects the gastrointestinal tract. CD is usually accompanied by other extragastrointestinal lesions, such as aphthous ulcers, uveitis, peripheral arthritis, or perianal abscesses that are easily confused with intestinal BD (4). Currently, there are no available diagnostic laboratory tests for either disease. Therefore, the diagnosis of intestinal BD and its differential diagnosis from CD is challenging for clinicians due to their similarities in intestinal and extraintestinal manifestations and pathological findings, especially for CD accompanied with BD-like extraintestinal manifestations (5). Some investigators speculate that the two disorders exist on a spectrum (6). Nevertheless, CD patients require corticosteroids or immunosuppressant therapies more often than intestinal BD patients (7). From the perspective of precision medicine, it is worthwhile to differentiate the two diseases. Precise diagnosis may aid the treatment, improving prognosis. Endoscopy is the first choice for clinicians to diagnose intestinal ulcers. Studies have shown that parameters including round ulcer, focal distribution, and cobblestone appearance in endoscopy are valuable for differentiating between intestinal BD and CD. In the present study, we retrospectively analyzed demographics, clinical manifestations, laboratory findings, disease activity, and endoscopic and pathological results. We compared the endoscopy characteristics of intestinal BD and CD. We found that intestinal BD and CD have overlapping manifestations, making it difficult to distinguish one from the other. The severity of intestinal mucosal injury in BD is slighter than that of CD. Endoscopy parameters are valuable for differentiating the two conditions. The differentiation model combining several endoscopy features appears to be reliable for distinguishing between intestinal BD and CD.

## 2. Materials and methods

### 2.1. Patients enrolled and exclusion criteria

We enrolled 861 patients with BD and 81 patients with CD admitted to Huadong Hospital, affiliated to Fudan University, between December 2012 and December 2017 consecutively. All patients with BD conformed to the International Study Group criteria for BD published in 1990, of which 111 (111/861, 12.9%) had ulcers objectively confirmed by endoscopy and met the Korean guidelines for diagnosing intestinal BD published in 2009 (8). Similarly, 81 patients who met the 2010 World Gastroenterology Organization Practice Guidelines for the diagnosis and management of inflammatory bowel disease were included as the CD group (9). Subjects with suspected appearance of any other gastrointestinal diseases such as intestinal tuberculosis, nonspecific colitis, or intestinal cancer were excluded. Patients who were taking nonsteroidal antiinflammatory drugs or other enterotoxic medications were excluded. Patients were also excluded if they had taken glucocorticoids or immunosuppressive agents during the previous month. Clinical data were collected after approval by the Institutional Ethics Committee, and informed consent was provided by all participants.

### 2.2. Clinical evaluation and data collected 

We reviewed general information, including sex, age, disease course, and clinical manifestations. Laboratory investigations included hemoglobin (Hb), C-reactive protein (CRP), and erythrocyte sedimentation rate (ESR). Ulcer characteristics on endoscopy included size (diameter ≥1 cm), shape, distribution, and number, as well as mucosal findings, pathological manifestations, and complications. The Simple Endoscopic Score of Crohn’s Disease (SES-CD) was used to evaluate the severity of intestinal mucosal injury.

### 2.3. Statistical analysis

Statistical analysis was performed using SPSS 17.0. The continuous variables were presented as mean ± standard deviation (SD). Categorical variables were presented as proportions. In a univariate analysis, continuous and categorical variables were analyzed by t‑test and χ2 test, respectively. All endoscopic predictors with P < 0.05 were entered into logistic regression for multivariate analysis to test whether a certain variable was independently associated. Six endoscopic variables were entered into the logistic regression model. The regression β coefficients were divided by the smallest coefficient and then rounded to the nearest integer to derive a risk score. We then calculated the risk score of the predictors for each patient. Finally, the differential diagnostic efficacy of the scoring system was tested by ROC curve. The significance level was set at P < 0.05. 

## 3. Results

### 3.1. Demographic and clinical manifestations of intestinal BD and CD

We recruited a total of 111 patients with intestinal BD (59 females and 52 males) and 81 with CD (32 females and 49 males) during the study period. There was slightly earlier onset and longer duration in intestinal BD patients than CD patients (P = 0.001 and P < 0.001, respectively). Intestinal BD presented more extraintestinal symptoms than did CD (P < 0.001). Oral ulcer was the most common presentation (97.3% [108/111]), followed by genital ulcer (74.77% [83/111]) and skin lesions (49.55% [55/111]). These signs were rare in CD patients. By contrast, gastrointestinal symptoms including abdominal pain, mucous feces, and hematochezia were seen more often in CD than in intestinal BD (P < 0.001) (Table 1).

**Table 1:** Comparison of the demographic and clinical manifestations between intestinal BD and CD.

Characteristics	Intestinal BD(n = 111)	CD(n = 81)	P-values
Demographics			
Sex (male:female)	1:1.13	1.53:1	0.061
Age at onset (years, mean ± SD)	33.79 ± 13.99	45.67 ± 17.05	0.001
Course (months, mean ± SD)	85.25 ± 62.19	47.78 ± 21.49	<0.001
Intestinal manifestations (n, %)			
Abdominal pain	47 (42.34)	55 (67.90)	<0.001
Diarrhea (>3 times/day)	31 (27.93)	43 (53.09)	<0.001
Mucous feces	9 (8.11)	39 (48.15)	<0.001
Hematochezia	14 (12.61)	33 (40.70)	<0.001
Extraintestinal manifestations (n, %)			
Oral ulcer	108 (97.3)	7 (8.64)	<0.001
Genital ulcer	83 (74.77)	0 (0)	<0.001
Erythema nodule/folliculitis	55 (49.55)	1 (1.2)	<0.001
Arthralgia	6 (17.8)	6 (7.3)	0.571
Ocular	7 (6.31)	0 (0)	0.056
Nervous	2 (1.80)	0 (0)	0.621
Hematological	8 (1.80)	0 (0)	0.036
Heart	1 (0.90)	0 (0)	1.000
Vascular	7 (6.31)	0 (0)	0.056

BD, Behçet’s disease; CD, Crohn’s disease; SD, standard deviation.

### 3.2. Laboratory results of patients with intestinal BD and CD

The differences in laboratory results for Hb (intestinal BD: 120.71 ± 20.61 g/L vs. CD: 119.54 ± 22.76 g/L, P = 0.329), ESR (intestinal BD: 32.70 ± 29.802 mm/h vs. CD: 36.11 ± 33.08 mm/h, P = 0.0.350), and CRP (intestinal BD: 29.89 ± 39.62 mg/L vs. CD: 32.54 ± 47.92 mg/L, P = 0.535) were not statistically significant. 

### 3.3. Endoscopic characteristics in intestinal BD and CD

#### 3.3.1. Endoscopic severity

Among 111 intestinal BD patients, there were 53 (47.75%) in remission, 37 (33.33%) with mild disease, 21 (18.92%) with moderate disease, and 0 (0.00%) with severe disease based on SES-CD scores. Among 81 CD patients, the number in remission and mild, moderate, and severe patients were 16 (19.75%), 27 (33.33%), 31 (38.27%), and 7 (8.64%), respectively. Moderate-to-severe endoscopic lesions were significantly more frequent in CD patients than in intestinal BD patients (P = 0.003 and 0.002, respectively). By contrast, the incidence of remission was significantly higher among intestinal BD patients than CD patients (P < 0.001) (Table 2).

**Table 2:** Comparison of the endoscopic severity between intestinal BD and CD.

SES-CD	Intestinal BD n (%)	CD n (%)	P-values
Remission (≤3)	53 (47.75)	16 (19.75)	<0.001
Mild (4–10)	37 (33.33)	27 (33.33)	0.563
Moderate (11–19)	21 (18.92)	31 (38.27)	0.003
Severe (≥20)	0 (0)	7 (8.64)	0.002

SES-CD, Simplified Endoscopic Score for Crohn’s Disease.

#### 3.3.2. Ulcer distribution and count

**Figure 1:**
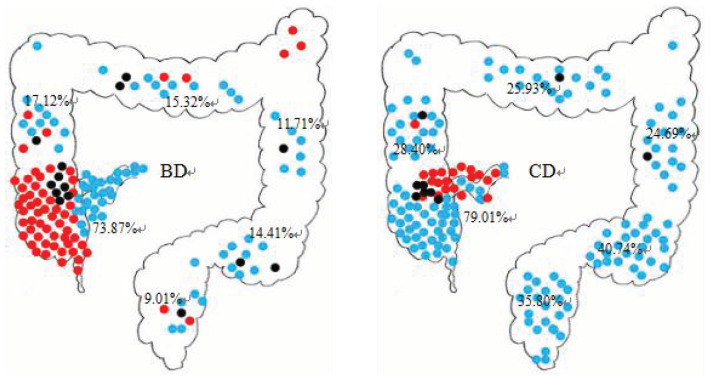
Distribution pattern and number of ulcers in intestinal BD and CD. Single ulcer: 1; oligo ulcers: 2–3; multiple ulcers: ≥4.

Ulcers can occur in the upper and lower digestive tract, and the incidences of ulceration in the gastroduodenal area (intestinal BD: 6.31% vs. CD: 22.22%, P = 0.001), descending colon (intestinal BD: 11.71% vs. CD: 24.69%, P = 0.019), sigmoid colon (intestinal BD: 9.01% vs. CD: 35.80%, P < 0.001), rectum (intestinal BD: 9.01% vs. CD: 35.80%, P < 0.001), and perianal area (intestinal BD: 0.90% vs. CD: 18.52%, P < 0.001) were significantly different. Perianal lesions were characterized by ulcers in intestinal BD, with the incidence rate of 2.7%. Perianal abscesses were characteristic features in CD patients, with an incidence higher than that of intestinal BD (P < 0.001) (Table 3). Although the ileocecal area is the most involved area for ulcers in both diseases (P = 0.410), solitary ulcers in the ileocecal area were more common in intestinal BD (P < 0.001). Multiple ulcers (>4 ulcers) often presented in CD (Table 3; Figure 1). Lesions in intestinal BD patients involved a single segment more often than did those in CD patients (P = 0.002). 

**Table 3:** Endoscopy findings in differential diagnosis between intestinal BD and CD.

Endoscopy findings	Intestinal BD n (%)	CD n (%)	P-value	
			Univariate analysis	Multivariate analysis
Distribution			
Esophageal ulcerations	8 (7.21)	4 (4.94)	0.521	
Gastroduodenal ulcerations	7 (6.31)	18 (22.22)	0.001	NS
Ileocecal ulcerations	82 (73.87)	64 (79.01)	0.410	
Ascending colon	19 (17.12)	23 (28.40)	0.062	
Transverse colon	17 (15.32)	21 (25.93)	0.068	
Descending colon	13 (11.71)	20 (24.69)	0.019	NS
Sigmoid colon	16 (14.41)	33 (40.74)	<0.001	NS
Rectum	8 (9.01)	29 (35.80)	<0.001	NS
Perianal abscess	1 (0.90)	15 (18.52)	<0.001	0.049
Single segment involved	82 (73.87)	29 (35.80)	0.002
<0.001	Solitary ulcer in ileocecal area	52 (46.85)	16 (19.75)	<0.001	<0.001
Diameter ≥1 cm	18 (16.22)	10 (12.35)	0.453	
Ulcerative shape				
Irregular shape	22 (19.82)	28 (34.57)	0.021	NS
Round/oval shape	62 (55.86)	20 (24.69)	<0.001	0.013
Stripe ulcer	3 (2.70)	20 (24.69)	<0.001	NS
NS	Annular ulcers	2 (1.80)	1 (1.23)	1.000	
Mucous hyperplasia				
Polyps	13 (11.7)	18 (22.22)	0.047	NS
Cobblestone sign	2 (1.80)	18 (22.22)	<0.001	NS
Complications				
Ileocecal valve malformation	31 (27.93)	19 (23.46)	0.486	
Perforation	9 (8.11)	5 (6.17)	0.870	
Intestinal obstruction	4 (3.60)	29 (35.80)	0.001	0.035
Intestinal fistula	3 (1.80)	14 (17.28)	<0.001	<0.001
Pathology				
Mucous inflammation	78 (70.2)	64 (79.0)	0.864	
Granulation tissue	24 (41.6)	30 (52.4)	0.143	
Vasculitis	1 (0.9)	1 (1.2)	0.059	

Ileocecal region represents terminal ileum and/or cecum.

#### 3.3.3. Ulcerative shape and mucous hyperplasia

**Figure 2:**
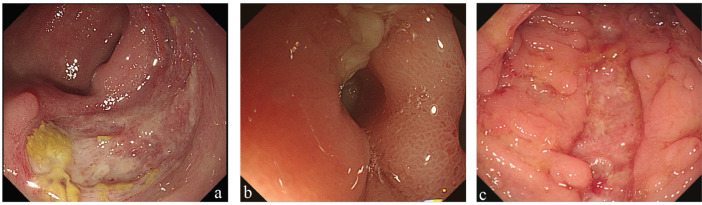
a) An isolated ulcer in the ileocecal region with mucosal hyperemia around the ulcer in a patient with intestinal BD. b) A longitudinal ulcer with ileocecal stenosis in a patient with CD. c) Diffuse longitudinal ulcer with cobblestone appearance in a patient with CD.

Intestinal BD more often presented round ulcers with mucosal hyperemia around the lesion (P < 0.001, Table 3; Figure 2a), whereas longitudinal ulcers and cobblestone appearance were found more frequently in CD than in intestinal BD patients (P < 0.001, Figure 2b). 

#### 3.3.4. Complications

Both diseases presented complications, including perforation, intestinal stenosis, and fistulas. Intestinal stenosis and fistula were more common in CD (P = 0.035 and P < 0.001, respectively). Other parameters, including diameter of ulcer and microscopic pathology, showed no differences between the two disorders (Table 3).

### 3.4. Multivariate analysis to determine the independent predictors and evaluation of the scoring model

**Figure 3:**
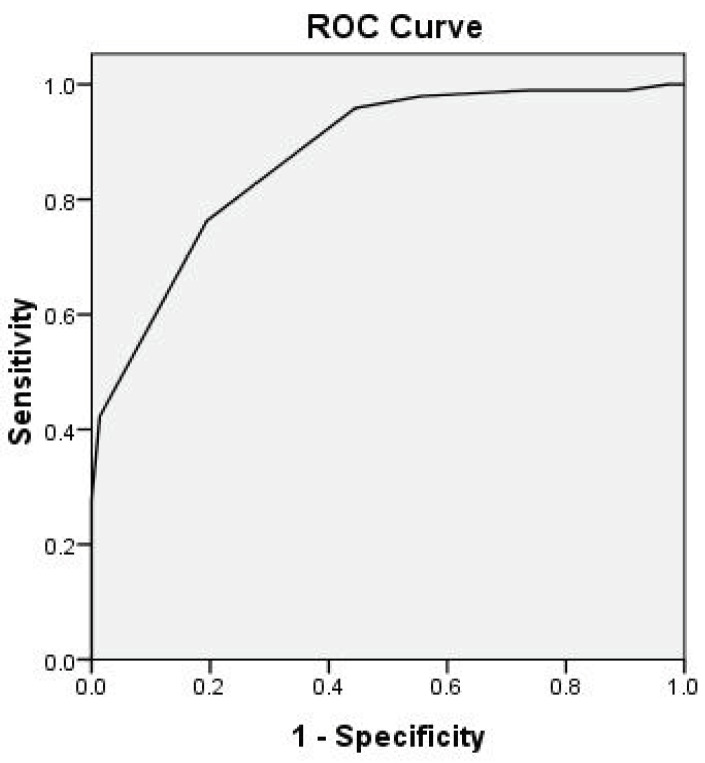
ROC curve of the differentiation model (area under the ROC curve is 0.874).

The potential indicators with P < 0.05 were entered into a binary logistic regression for multivariate analysis. Solitary ulcer in the ileocecal area, single segment involvement, and round-shaped ulcer were independent predictors of intestinal BD (P < 0.001, P < 0.001, and P = 0.013, respectively), whereas perianal abscess, intestinal obstruction, and fistula were independent predictors of CD (P = 0.049, P = 0.035, and P < 0.001, respectively). The β-coefficient of round-shaped ulcers was the smallest. The β-coefficients for the other variables were divided by the minimum regression coefficient, then rounded to the nearest integer as the scores of each variable. The scores ranged from –2 to 3 (Table 4). The area under the ROC curve (95% CI) was 0.874 (0.823–0.926, Figure 3), which indicating that the scoring system showed good discrimination.

**Table 4:** Multivariate analysis of endoscopic findings to distinguish intestinal BD from CD

Endoscopy findings	Β-value	P-value	95% CI	Score	
				YES	NO
Solitary ulcer in ileocecal	–2.322	<0.001	0.032-0.302	2	0
Perianal abscess	2.890	0.049	1.008–321.275	–2	0
Single segment	–1.902	<0.001	0.054–0.415	1	0
Round shape	–1.290	0.013	0.099–0.762	1	0
Intestinal obstruction	1.642	0.035	1.122–23.810	–1	0
Fistula	3.449	<0.001	5.902–167.748	3	0

Ileocecal region represents terminal ileum and/or cecum.

## 4. Discussion

BD was originally reported by Turkish dermatologist Hulusi Behçet in 1937. It is generally considered to be a multifactorial disease, characterized by recurrent oral aphthous ulcers, genital ulcers, and uveitis. Sometimes BD patients can present with gastrointestinal ulcers at sites and with clinical manifestations resembling those of CD. It is easily misdiagnosed because there are no specific pathological or laboratory markers for the diagnosis of the entity. Several case reports and clinical studies highlighted the difficulties in making this distinction (5,10–13). To date, several discriminating endoscopic findings have been reported, including round shape, five or fewer lesions, focal distribution, and absence of aphthous and cobblestone lesions as features supporting the diagnosis of intestinal BD (12). Our study confirmed that there were significant differences to these five predictors. We also found that intestinal obstruction and fistula were two additional independent discriminating predictors. In addition to the differences on endoscopy, Li et al. (11) found that massive gastrointestinal hemorrhage, fever, and extraintestinal manifestations were significantly more common in intestinal BD, while diarrhea, intestinal obstruction, and perianal abscess were more common in CD. Nevertheless, clinical manifestations are subjective and easily influenced by recall bias during a long disease course. Thus, the aim of this article was to stress the value of endoscopy features and to establish a differential diagnosis scoring system with combined multiple signs of endoscopy. 

Studies found that over half of intestinal BD patients were misdiagnosed as having CD at their first visit. Significant clinical similarities may contribute to the high rate of misdiagnosis (10). Consistent with past experience, there are factors leading to misdiagnosis between intestinal BD and CD. Both diseases have a young age of onset and a long course. Extraintestinal manifestations can be present in both diseases, including recurrent oral ulcers, skin lesions, and arthritis, as well as gastrointestinal symptoms such as abdominal pain, diarrhea, mucous feces, and hematochezia. Furthermore, both diseases are widely distributed throughout the upper and lower alimentary tract, and the involvement of the ileum is most common in both intestinal BD patients (73.87%) and CD patients (79.01%). However, there are many differences between intestinal BD and CD on careful analysis. 

Research has historically suggested that mucocutaneous lesions were the most powerful discriminating factors between the diseases (11). Our study confirmed that oral ulcers, genital ulcers, mucocutaneous lesions, and hematological disease were suggestive factors for intestinal BD on univariate analysis. CD may also be characterized by oral ulcers, joint pain, and erythematous nodules (4); however, there are no more than two extraintestinal symptoms in one CD case. Digestive symptoms of intestinal BD are similar to those of CD. These include abdominal pain, diarrhea, and mucosanguineous feces; however, the incidence was significantly lower than that of CD. This suggests that mucosal inflammation in patients with intestinal BD was milder than that of CD. Intestinal symptoms usually occur on average 4.5–6 years after the onset of oral ulcers (14). Our statistical result was 85.25 ± 62.19 months, significantly longer than the course in CD patients, possibly associated with a low incidence of intestinal symptoms in the early diagnosis of BD. By routine endoscopy we found that the incidence of asymptomatic patients in intestinal BD was 62.86% (3). Therefore, clinicians often ignore the presence of ulcerative lesions when BD patients present to a physician (15).

Enteric ulcers in both disorders can occur in any part of the alimentary tract, with the ileocecal region being predominantly affected (11). The mucosal damage in patients with intestinal BD is lighter than in those with CD, possibly explaining why digestive symptoms are rare in patients with intestinal BD. These lesions are primarily characterized by the following aspects: first, consistent with the results of another study (16), lesions in intestinal BD patients tended to be solitary ulcers, especially in the ileocecal segment; second, single segment involvement was more common in intestinal BD patients than in CD patients (73.87% vs. 35.80%, P < 0.001). By contrast, ulcers in CD were usually widespread, but not confined to the ileocecal region. Gastroduodenal, descending colon, sigmoid colon, and rectum involvement were more frequently present in CD patients than in intestinal BD patients (P = 0.001, P = 0.019, P < 0.001, and P < 0.001, respectively); third, mucosal hyperplasia including pseudopolyps and cobblestone appearance, resulting from repeated inflammation and ulceration associated with excessive healing processes, were often absent in intestinal BD (17); fourth, the incidence of complications including intestinal obstruction and fistula in patients with intestinal BD was lower than that of CD. In addition, there were differences between the two disorders in terms of ulcer shape and perianal lesions. Round or oval shape, longitudinal ulcers, and perianal fistulas or abscesses were also discriminating predictors. These findings were consistent with those of Li et al. (11) and Zhang et al. (17). 

Because the incidence of each index in a disease is very low, differentiating between these two conditions with a single parameter is difficult. Therefore, the establishment of algorithms, comprehensive analyses of endoscopic results, and combination with clinical history is helpful for diagnosis. In 2009, Lee et al. generated algorithms based on colonoscopy and found that more than 90% of cases could be diagnosed by the algorithm (12). In the present study, we found that the distinguishing markers of intestinal BD were solitary ulcer in the ileocecal area, single segment involvement, and round-shaped ulcer, whereas distinguishing markers of CD were longitudinal ulcers, intestinal obstruction, and fistulas. Finally, six endoscopic parameters were entered into a logistic regression model to establish the scoring model. Verification was performed within the dataset with ROC curves. The area under the ROC curve was 0.874 (95% confidence interval: 0.823–0.926, P < 0.001), suggesting that the scoring system was highly reliable for differentiating the two diseases and that it was convenient for use by clinicians. The sensitivity and specificity were 73.2% and 84.7%, respectively, for a score greater than 1.5 in the diagnosis of intestinal BD, indicating that the reliability of the scoring system was acceptable.

This study has some limitations: first, its retrospective nature and limited number of patients carry the possibility of selection bias; second, as the number of cases was small, we verified the scoring model with the original dataset. We expect further studies with larger samples and more prospective studies being carried out to verify this conclusion. Third, this differentiating model could be used only if other diseases have been excluded, possibly limiting its application. 

In conclusion, intestinal BD and CD have differences in terms of clinical and endoscopy features. Extraintestinal manifestations primarily occurred in intestinal BD, while the symptoms of the intestine were not substantial, possibly related to the mild injury of the intestinal mucosa. The model established according to endoscopy parameters appeared to be reliable for differentiating between intestinal BD and CD. The scoring model could be conveniently used by clinicians. 

## Acknowledgments

This work was supported by the Clinical Science Innovation Program of Shanghai Shenkang Hospital Development Center (SHDC12017129).
